# Dr. Andrew J. Butler

**Published:** 1916-04

**Authors:** 


**Dr. Andrew J. Butler,** of Unadilla, Rush 1891, cut his throat with a razor March 3. He was exhausted and probably temporarily out of his mind, on account of an all-night vigil with a patient.

